# Genetic Relationships Among Physiological Processes, Phenology, and Grain Yield Offer an Insight Into the Development of New Cultivars in Soybean (*Glycine max* L. Merr)

**DOI:** 10.3389/fpls.2021.651241

**Published:** 2021-04-09

**Authors:** Miguel Angel Lopez, Fabiana Freitas Moreira, Katy Martin Rainey

**Affiliations:** ^1^Colombian Sugarcane Research Center, Cali, Colombia; ^2^Bayer CropScience (United States), St. Louis, MI, United States; ^3^Department of Agronomy, Purdue University, West Lafayette, IN, United States

**Keywords:** photosynthesis, water use efficiency, stability, relative contribution, physiological breeding, unsupervised method

## Abstract

Soybean grain yield has steadily increased during the last century because of enhanced cultivars and better agronomic practices. Increases in the total biomass, shorter cultivars, late maturity, and extended seed-filling period are frequently reported as main contributors for better soybean performance. However, there are still processes associated with crop physiology to be improved. From the theoretical standpoint, yield is the product of efficiency of light interception (*Ei*), radiation use efficiency (*RUE*), and harvest index (*HI*). The relative contribution of these three parameters on the final grain yield (*GY*), their interrelation with other phenological–physiological traits, and their environmental stability have not been well established for soybean. In this study, we determined the additive–genetic relationship among 14 physiological and phenological traits including photosynthesis (*A*) and intrinsic water use efficiency (*iWUE*) in a panel of 383 soybean recombinant inbred lines (RILs) through direct (path analyses) and indirect learning methods [least absolute shrinkage and selection operator (LASSO) algorithm]. We evaluated the stability of *Ei*, *RUE*, and *HI* through the slope from the Finley and Wilkinson joint regression and the genetic correlation between traits evaluated in different environments. Results indicate that both supervised and unsupervised methods effectively establish the main relationships underlying changes in *Ei*, *RUE*, *HI*, and *GY*. Variations in the average growth rate of canopy coverage for the first 40 days after planting (*AGR40*) explain most of the changes in *Ei*. *RUE* is primarily influenced by phenological traits of reproductive length (*RL*) and seed-filling (*SFL*) as well as *iWUE*, light extinction coefficient (*K*), and *A*. *HI* showed a strong relationship with *A*, *AGR40*, *SFL*, and *RL*. According to the path analysis, an increase in one standard unit of *HI* promotes changes in 0.5 standard units of *GY*, while changes in the same standard unit of *RUE* and *Ei* produce increases on *GY* of 0.20 and 0.19 standard units, respectively. *RUE*, *Ei*, and *HI* exhibited better environmental stability than *GY*, although changes associated with year and location showed a moderate effect in *Ei* and *RUE*, respectively. This study brings insight into a group of traits involving *A*, *iWUE*, and *RL* to be prioritized during the breeding process for high-yielding cultivars.

## Introduction

Through the combined contribution of breeding, agronomy, and climate change, soybean yield has achieved a dramatic improvement. A steady yield increase of 24.7 kg ha^–1^ year^–1^ (Specht et al., 2014; USDA–NASS, 2020) has almost quintupled productivity compared with the 740 kg ha^–1^ produced in 1924. Retrospective studies showed that breeding and agronomy have effectively contributed to a relatively similar percentage to the soybean yield improvement during the last decades (Specht and Williams, 1984; Specht et al., 1999). Contribution of increased CO_2_, also called carbon fertilization, is based on the stimuli in the net carbon fixation in species C3 via better control of photorespiration (Specht et al., 1999; Ainsworth et al., 2012; Taiz et al., 2014). Variation on productivity as a result of CO_2_ increase has been estimated in a wide interval from 4.3 to 32.0% with a likely contribution in the range of 5-10% (Specht et al., 1999; Ainsworth et al., 2012; Sakurai et al., 2014).

Through changes guided by genetic, breeding, and market, soybean went from being considered a forage crop using plant introduction from East Asia in the early 1900s to the adoption of bred cultivars with better adaptation to North America in 1940 (Hartwig, 1973; Rincker et al., 2014). Selection for yield was the first target and later complemented with pest resistance, while proprietary breeding programs joined public efforts at the level of currently providing most of the soybean seed required for farmers in North America (Carter et al., 2004; Specht et al., 2014). Breeding strategies have focused on optimizing plant structure and seed composition. New cultivars are frequently shorter, are less prone to lodging and shattering, mature later, and also produce more branches and more pods from these branches especially under low density (Specht and Williams, 1984; Carter et al., 2004; Evans and Sadler, 2008; Fox et al., 2013; Rincker et al., 2014; Suhre et al., 2014). Improvements in canopy along with an extended seed-filling length led to greater solar radiation capture during this developmental stage (Boerma and Ashley, 1988; Kumudini et al., 2001; Koester et al., 2014). Augmented total dry matter production has contributed heavily in better yielding regardless of the mixed reports about increased or constant dry matter partition to the seeds (Kumudini et al., 2001; Rowntree et al., 2014; Balboa et al., 2018). Modern cultivars also incorporated resistance to pest and disease reducing potential losses (Johnson, 1987; Heatherly and Elmore, 2004; De Bruin and Pedersen, 2009). Breeding achievements also involved transgenic soybean and resistance to glyphosate, which, since 1996, changed the weed control, making it more flexible, simpler, and opportune (Reddy, 2001). Seed composition and yield components have been optimized to meet new requirements for industry and human health (Morrison et al., 2000; Ustun et al., 2001). While protein concentration was reduced, oil concentration and oil composition were increased, favoring monounsaturated fat acids (oleic) (Wilcox et al., 1979; Morrison et al., 2000; Ustun et al., 2001; Giannakas and Yiannaka, 2004; Rowntree et al., 2013; Rincker et al., 2014). Increase in seed weight is not always consistent or, if positive, less than 0.10 g per 100 seeds, suggesting bigger contribution to increased yield from more seeds per plant, or more plant per hectare (Specht and Williams, 1984; Voldeng et al., 1997; Morrison et al., 2000; Wilson et al., 2014).

Agronomy has also contributed to a better soybean performance through new or enhanced technologies, techniques, and practices. Remarkable changes started during the first four decades of the last century when animal power was replaced by tractors, the mechanical harvesters were introduced, and the shift in vocation from forage to protein-oil crop occurred (Probst and Judd, 1973; Bogue, 1983; Gardner, 2002; Egli, 2008). Later, improvements associated with earlier planting date (Johnson, 1987; Specht et al., 1999; Bastidas et al., 2008; Suhre et al., 2014), reduced row spacing (Voldeng et al., 1997; Cregan et al., 1999; Heatherly and Elmore, 2004), higher seeding rates (Voldeng et al., 1997; De Bruin and Pedersen, 2009), reduced harvest losses (Johnson, 1987; Ustun et al., 2001), better crop nutrition through fertilizer and crop rotation (Luedders, 1977; Wilson et al., 2014; Grassini et al., 2015), and, in general, superior control of factors producing biotic or abiotic stress (Egli, 2008; Suhre et al., 2014) have facilitated to exploit the genetic yield potential.

Theoretical calculations indicate soybean grain yield potential is around 8,000 kg ha^–1^ (Specht et al., 1999). However, the current yield is still quite far from this potential with 3,409 kg ha^–1^ in 2020 (Specht et al., 2014; USDA–NASS, 2020). Although closing the gap is a common effort involving not only plant breeding but also better agronomic practices, a clear identification of factors or traits to be prioritized must be carried out to concentrate efforts and resources. From the physiological standpoint, potential grain yield is the product of efficiencies accounting for the capture and transformation of solar radiation into biomass abbreviated, respectively, as *Ei* and *RUE*, and the later efficiency of allocation of dry matter to the economically important organs or *HI* (Monteith, 1972, 1977). In soybean, although studies involving one or more of these three efficiencies are available with particular focus on *HI* (Shibles and Weber, 1966; Spaeth et al., 1984; Board and Harville, 1993; Kumudini et al., 2001; De Bruin and Pedersen, 2009; Fox et al., 2013; Koester et al., 2014; Rowntree et al., 2014; Suhre et al., 2014), the influence of other physiological and phenological variables on *Ei*, *RUE*, and *HI* as well as the interrelation among this three efficiencies and their partial contribution to *GY* is not documented in soybean.

Determining the relationship among these agronomical, physiological, and phenological variables requires the implementation of multivariate methodologies where genetic and environmental relationships are established. Classical approach to establish interrelation among variables include the supervised path analysis method, where a set of lineal equations are defined based on a correlation matrix and theoretical background (Wright, 1960; Bondari, 1990; Walsh and Lynch, 1998). Path coefficients provide more information than traditional correlations since they not only present the partial contribution of predictors on the response variables but also report direct and indirect effects (Bondari, 1990; Board et al., 1999). Unsupervised machine learning methods offer new alternatives to establish complex interactions among variables through undirected graphical models (Hastie et al., 2009; Steinsland and Jensen, 2010). An example is the Markov network machine learning method, which does not require specificity for direction and is suitable for spatial or relational data for uncovering variable structure and dependence (Murphy, 2014). Previous studies to establish interrelations among agronomical and phenological variables have been already performed, and works through historical panels have also indirectly approached these relationships (Specht et al., 1999; Morrison et al., 2000; Rincker et al., 2014; Suhre et al., 2014; Xavier et al., 2017a). Directed and undirected methods in soybean have been independently reported by Board et al. (1999) and Xavier et al. (2017a), focusing on, in the first case, yield components and, in the second, phenology, canopy development, and yield component. However, these studies lack the inclusion of physiological processes and efficiencies accounting for changes in the potential yield such as *Ei*, *RUE*, *A*, and *iWUE*. In addition, comparison of result from these two methods in soybean is not reported.

In this study, we established the genetic correlations among agronomical, physiological, and phenological variables and the three efficiencies controlling the potential grain yield in soybean: efficiency of light interception, radiation use efficiency, and harvest index (Monteith, 1972, 1977). Likewise, we determined the relative contribution of *Ei*, *RUE*, *HI*, and other physiological variables as *A*, and *iWUE* to the *GY* in soybean through direct (path analysis) and undirected graphical model [least absolute shrinkage and selection operator (*LASSO*) algorithm] methodologies based on additive–genetic variance–covariance matrices. Finally, we evaluated the stability of *Ei*, *RUE*, and *HI* using the genetic correlations between the same trait evaluated in a different environment and the slope from the Finlay and Wilkinson joint regression (FWR). This paper suggests traits to be prioritized during the breeding process as a strategy to improve the grain yield in soybean.

## Materials and Methods

### Plant Material and Experimental Design

A maturity-controlled panel of 383 recombinant inbred lines (RILs) selected from the Soybean Nested Association Mapping collection SoyNAM was used. A panel was selected constraining maturity and evaluated as days required to get *R8* (Fehr and Caviness, 1977), while retaining variation to *GY* (Supplementary Figure 1). Thus, the maturity group for these indeterminate RILs was similar and considered as group 3 MG III. RIL selection was performed using as data set field data collected during the seasons 2011 and 2014 in Indiana and Illinois. These 383 RILs come from 32 families classified into three main classes according to the type of cross originally made: high yielding (*HY*), high yielding under drought conditions (*HYD*), and diverse ancestry (*DA*). A complete description of families, crosses, and extra information is available in^1^ and Lopez et al. (2019), while the complete list of families and RILs is presented in Supplementary Tables 1, 2. Three environments were considered for this study, which correspond to the combination of location × year. An experimental design alpha lattice incomplete block design, with two complete replications and 32 incomplete blocks per replication, was planted in the location ACRE (40°28′20.5′′N 86°59′32.3′′W) at West Lafayette, IN, during 2017 (ACRE_2017). The same experiment was implemented in Romney, IN (40°14′59.1′′N 86°52′49.4′′W—RMN_2018), and ACRE again (ACRE_2018) during 2018. The experimental unit corresponded to six-row plots (0.76 m × 3.35 m) planted with a target population of 35 seed m^–2^. Plots with non-uniform emergence were discarded, reducing the number of RILs to 322 for ACRE_2017 and 381 for RMN_2018. Soil types for ACRE included Chalmers silty clay loam (Typic Endoaquolls) and Raub–Brenton complex (Aquic Argiudolls), while RMN corresponded to Drummer soils (Typic Endoaquolls) (NRCS, 2018). High natural soil fertility was confirmed through the soil analysis (Supplementary Table 3), which, along with the crop management, ensured adequate nutritional status during the growing season. Although it was a rainfed study, water was not a limiting factor as confirmed by the water balance (Supplementary Tables 4–6). The main environmental characteristics are summarized in Table 1.

**TABLE 1:** Main environmental characteristics for each location.

Characteristic	ACRE_2017	ACRE_2018	RMN_2018
Soil type	Raub–Brenton complex	Chalmers silty clay loam	Drummer
Field capacity % by weight	28.5	27.6	25.9
Permanent wilting point % by weight	13.3	12.4	12.4
Bulk specific density, g cm^–3^	1.3	1.4	1.4
Cumulative rainfall mm	487.7	458.2	346.5
Average rainfall, mm month^–1^	132.0	130.0	91.0
Cumulative solar radiation, MJ m^–2^	2,038.6	1,899.1	2,128.5
Mean air temperature, °C	21.4	23.4	23.5
Maximum air temperature, °C	28.2	29.6	29.4
Minimum air temperature, °C	15.1	17.5	18.0
Cumulative growth degree days	1,270.6	1,431.9	1,452.0
Reference evapotranspiration, mm/day	2.4	2.3	2.5
Days to emergence	9	7	9

### Phenotypic Traits

A fixed-wing *UAV*-type eBee equipped with an S.O.D.A red–green–blue (RGB) camera (senseFly Parrot Group, Switzerland) was flown with a frequency of ∼12 days. Canopy coverage (*CC*) was obtained from the RGB imagery through the software Progeny^®^ (Progeny Drone Inc., West Lafayette, IN) using a multilayer mosaic approach as described by Hearst (2019). Aboveground dry matter for each plot was sampled during the growing season in a linear section of 0.56 m in a row with perfect competence. The fresh biomass collected from each sampling site was dried at 80°C using a dry air system until constant weight. Three full biomass samplings (∼38, 58, and 84 days after planting – *DAP*) were considered for both environments in 2018, while just one sampling when maximum biomass accumulation was achieved at 91 *DAP* was carried out in 2017. Biomass was adjusted through a linear model involving RIL, environment, and replication as variables and number of plants as a covariate to avoid potential differences in biomass due to the number of plants. Seed weight was directly calculated from a lineal sample size of 0.56 m harvested and threshed at maturity (*R8*) (Fehr and Caviness, 1977). *Ei* was calculated as the simple ratio between the solar radiation intercepted by the canopy and the total photosynthetic active radiation (*PAR*) available. To determine the daily solar radiation intercepted, a series of 766 logistic models, one per plot, were fitted following equation (1) through the R software (R Core team, 2019) package “*growthrates*” (Petzoldt, 2018). We used the *CC* as a proxy for light interception considering the direct relationship between these two parameters previously documented by Purcell (2000) and Xavier et al. (2017b).

(1) $$y=\frac{k*y\,0}{y\,o+\left(k-y\,o\right)*e\,x\,p^{-\mu \,m\,a\,x*t\,i\,m\,e}}$$

where *y* is canopy coverage, *yo* is the minimum canopy coverage value measured, *k* corresponds to the maximum canopy value or load capacity, *μmax* is the maximum relative growth rate, and *time* indicates days after planting.

Radiation use efficiency in 2018 environments was calculated as the slope of a linear regression between the total dry matter aboveground and the cumulative *PAR* intercepted. In 2017, since only one biomass sampling was performed, a simple ratio between the total aboveground dry matter and the cumulative *PAR* intercepted was used. Apparent *HI* was calculated as the direct ratio between the seed weight (0% moisture) and the total aboveground dry matter. Grain yield was determined in two perfect competence rows from each plot through a mechanical harvest. The weight registered was adjusted to 13% seed moisture and extrapolated to the hectare. Phenological stages *R1*, *R5*, and *R8* corresponding to days required to achieve flowering, beginning of seed, and maturity were scored three times per week following the criteria presented by Fehr et al. (1971). Length of the reproductive period was obtained by subtracting days to *R8* to days to *R1*, while seed-filling length was calculated as *R7* minus *R5* in days.

*AGR40* was measured as the mean of the daily growth rate during the first 40 days after planting. Growth rate corresponds to the first derivative from each logistic model adjusted for *CC*. Photosynthesis and intrinsic water use efficiency were measured through a portable photosynthesis system (LI-COR 6400XT, LI-COR, Lincoln, NE) set with a *PAR* value of 1,600 μmol photons m^–2^ s^–1^. CO_2_ concentration, temperature, and relative humidity were controlled to be 400 μmol mol^–1^, 25°C, and 75 ± 10%, respectively. The gas exchange parameters were measured before the seed filling phenological period, from late *R4* and early *R5* (Fehr and Caviness, 1977), in the third uppermost fully developed leaf, in three representative plants from each experimental unit from a complete replication. Additional details about the gas exchange protocol and measurements are available in Lopez et al. (2019).

Maximum leaf area index was recorded in a single measurement when the full canopy was achieved (60–70 *DAP*). A portable canopy analyzer (LI-2200, LI-COR, Lincoln, NE) following the protocol for small plots in row crops suggested by LICOR (LI-COR Inc, 2012) was used. Light extinction coefficient (*K*) was calculated through the light attenuation within a canopy theory reported by Monsi and Saeki (1953). Maximum *LAI* along with light measurements above and below the canopy was considered following equation (2):

(2) $$I=I_{0}\,e^{-K*L\,A\,I}$$

where *I* is the photosynthetic photon flux density (PPFD) measured on a horizontal plane, *LAI* is the leaf area index cumulated from top of the canopy, and *K* is the extinction coefficient. *I*_0_ is the PPFD above the canopy.

### Genetic Correlations

Best linear unbiased estimator (*BLUE*) per environment was calculated through a mixed model approach through the “*lme4*” package (Bates et al., 2015) in the software R following the statistical model below:

(3) $$Y_{i\,j\,k}=\mu +f\,\left(x\right)+\alpha_{i}+{\left(\alpha \,\beta \right)}_{i\,j}+\delta_{k}+e_{i\,j\,k},$$

where *Y* is the vector of phenotypes measured in the *i*^*th*^ replication, into the *j*^*th*^ block for the *k*^*th*^ RIL. μ is the intercept, *f*(*x*) controls the spatial heterogeneity within replications, α accounts for the effect of replication, *αβ* corresponds to the interaction replication × block, δ accounts for the genetic effect, and *e* controls the error. The covariate *f*(*x*) was computed as the average phenotypic value from the four closer surrounding plots (Lado et al., 2013) through the function *NNsrc* from the R “*NAM*” package (Xavier et al., 2015). In this model, the spatial covariate and the RILs were treated as fixed effects, while the other sources of variation were considered as random with any random effect_*r*_ ∼ N(0, σ^2^_*r*_), and *e* ∼ MVN(0, R).

*BLUE*s standardized by environment for all the traits were used to fit a second mixed model in a multivariate approach through the function *reml* in the “*NAM*” R package (Xavier et al., 2015). Additive–genetic effects were accounted for in this second model through a kinship matrix generated from a set of 23,119 single-nucleotide polymorphisms (SNPs) (Lopez et al., 2019). From this multivariate mixed model, two variance–covariance matrices were produced, G and R, where G corresponds to the additive–genetic matrix and R (residual) resembles the environmental relationships since *BLUE* values were used as input data. Correlations were calculated following the standard formula using the covariance between traits as the numerator and the product of their standard deviation as the denominator.

### Path Analysis, Unsupervised Model, and Environmental Trait Stability

A path analysis using the additive–genetic correlation derived from the G matrix was carried out to calculate the standardized path coefficients through the R package latent variable analysis “*lavaan*” (Rosseel, 2012) followed by a graphical representation through the R package “*semplot*” (Epskamp et al., 2019). Likewise, we implemented an undirected graphical model based on the same G matrix to establish the connection among traits. A Gaussian undirected graphical model based on neighborhood selection with the *LASSO* algorithm (Meinshausen and Bühlmann, 2006) implemented in the R package “*huge*” (Zhao et al., 2012). Finally, environmental stability for *Ei*, *RUE*, and *HI* was evaluated through two methodologies: (1) as the additive–genetic correlation between the same traits measured in the three different environments and (2) through the slope of the FWR (Finlay and Wilkinson, 1963). The Kendall correlation is used rather than the Pearson correlation, since Kendall assesses statistical association based on ranking (Kendall, 1938); thus, a positive correlation means that when the rank of certain trait evaluated in one environment increases, the rank of the same trait evaluated in another environment also increases. Kendall correlations were evaluated using the software R following formula (4):

(4) $$\tau =\frac{\begin{array}{c} \left(n\,u\,m\,b\,e\,r\,o\,f\,c\,o\,n\,c\,o\,r\,d\,a\,n\,t\,p\,a\,i\,r\,s\right)\\ -\left(n\,u\,m\,b\,e\,r\,o\,f\,d\,i\,s\,c\,o\,r\,d\,a\,n\,t\,p\,a\,i\,r\,s\right) \end{array}}{n\,\left(n-1\right)/2}$$

where τ indicates the Kendall correlation and *n* is the number of observations.

Where FWRs were implemented through the “*FW*” package in R under a Bayesian approach (Lian and de los Campos, 2016; Kusmec et al., 2017; Vanous et al., 2019). A nongenomic relationship matrix was used during the implementation; then Ad = I, where I is the identity matrix. RILs with missing information for one or more environments were discarded for this analysis. Slopes from FWR assess stability using the phenotypic values corrected by replication and incomplete block as input where all the genetic effects are presented, whereas correlations use the breeding values where only additive genetic effects are considered.

## Results

High positive additive–genetic correlations were identified for *Ei* with *AGR40* and *K*, contrasting with a negative correlation found between *Ei* and *R8*, *SFL*, and *RUE* (Table 2). Narrow-sense heritability for *Ei* reported a value of 0.65. Harvest index was positively correlated with *GY*, *A*, *R8*, and *RL*, while negatively correlated with *R5*. *HI* heritability was similar to *Ei* with 0.68. *RUE*, in turn, showed a moderated additive correlation with *RL*, *R1*, *K*, and *AGR40*, while its heritability was calculated to be 0.36. *GY* was positively associated with *RL*, *R8*, and *A*, while negatively correlated with *R1* and *R5*. Narrow-sense heritability for *GY* corresponded to 0.82. Other high genetic correlations include *AGR40* with *K*, *RL* with *R1*, and *R8* (Table 2). The descriptive statistics of mean, maximum, and minimum for the traits here considered are reported in Table 3.

**TABLE 2:** Additive–genetic correlation and narrow-sense heritability (diagonal) from a multitrait mixed model for physiological and phenological variables in a maturity-controlled panel of soybean.

Trait	*Ei*	*HI*	*GY*	*RUE*	*AGR40*	*R8*	*R1*	*R5*	*RL*	*SFL*	*LAI*	*K*	*A*	*iWUE*
***Ei***	0.65													
***HI***	0.07	0.68												
***GY***	0.11	0.62	0.82											
***RUE***	–0.35	–0.14	0.20	0.36										
***AGR40***	0.94	0.11	0.12	–0.27	0.71									
***R8***	–0.41	0.48	0.49	0.16	–0.33	0.69								
***R1***	0.12	–0.32	–0.61	–0.35	0.05	–0.26	0.78							
***R5***	–0.08	–0.55	–0.41	0.13	–0.05	0.17	0.55	0.71						
***RL***	–0.28	0.50	0.71	0.31	–0.19	0.73	–0.85	–0.29	0.84					
***SFL***	–0.47	0.30	0.29	0.07	–0.43	0.69	–0.18	0.15	0.51	0.61				
***LAI***	0.23	–0.10	0.30	–0.09	0.30	0.26	–0.02	0.30	0.16	–0.27	0.56			
***K***	0.67	–0.04	–0.05	–0.33	0.64	–0.29	0.02	–0.05	–0.16	–0.57	0.37	0.43		
***A***	–0.03	0.72	0.43	–0.12	–0.08	0.09	–0.27	–0.63	0.24	0.06	–0.18	–0.31	0.20	
***iWUE***	0.02	–0.18	–0.02	0.25	0.11	–0.36	–0.23	–0.43	–0.05	–0.30	–0.14	–0.19	0.12	0.20

*Three hundred eighty-one recombinant inbred lines (RILs) evaluated in three environments. *A*, photosynthesis; *AGR40*, average canopy coverage growth rate during the first 40 *DAP*; *Ei*, efficiency of light interception; *GY*, grain yield; *HI*, harvest index; *K*, light extinction coefficient; *iWUE*, intrinsic water use efficiency; *LAI*, leaf area index; *R1*, days to flowering; *R5*, days to beginning of seed formation; *R8*, days to maturity; *RL*, reproductive period length; *RUE*, radiation use efficiency; *SFL*, seed-filling length.*

**TABLE 3:** Descriptive statistic for the evaluated phenological and physiological traits.

Trait	ACRE_17	ACRE_18	RMN_18
*Ei* (%)	56.3 (50.7–59.4)	52.8 (47.2–57.9)	44.5 (36.7–50.2)
*RUE* (g MJ^−1^)	1 (0.6–1.2)	1.2 (0.7–1.5)	1.3 (0.8-1.5)
*HI*	0.4 (0.3–0.5)	0.4 (0.2–0.5)	0.4 (0.3–0.5)
*GY* (kg ha^−1^)	3178.2 (1587.4–4768.1)	3793.2 (2230.2–5159.8)	3886.1 (2504.7–5341.7)
*AGR40* (%/day)	1.2 (0.9–1.4)	0.9 (0.7–1.1)	0.6 (0.3–0.9)
*R8* (days)	114.6 (108.8–121)	121.6 (115.2–129.2)	124.9 (117.9–132.2)
*R1* (days)	39.1 (33.9–46)	39.4 (33.8–46.3)	41.7 (36.2–47.8)
*R5* (days)	67.5 (65.8–69.8)	74.2 (72.4–76.5)	77.3 (75.7–79.8)
*RL* (days)	75.5 (65–85.1)	82.1 (70.2–91.5)	83 (72-91.9)
*SFL* (days)	42.3 (35.7–47.1)	39.1 (33–43)	39.9 (34–44.1)
*LAI* (m^2^ m^−2^)	5.3 (2.7–7.6)	4 (1–7.3)	5.7 (3.3–8.1)
*K*	0.6 (0.8–0.3)	0.4 (0.7–0.3)	0.4 (0.6–0.2)
*A* (μmol CO_2_ m^−2^ s^−1^)	27 (23–30.6)	27.3 (22.3–31.8)	26.6 (21.3–30.4)
*iWUE* (μmol CO_2_ mol^−1^ H_2_O)	20.2 (0.6–54.4)	17.1 (0.6–37.3)	22.7 (2–48.2)

*Numbers in parenthesis show the range for each variable.*

**FIGURE 1:**
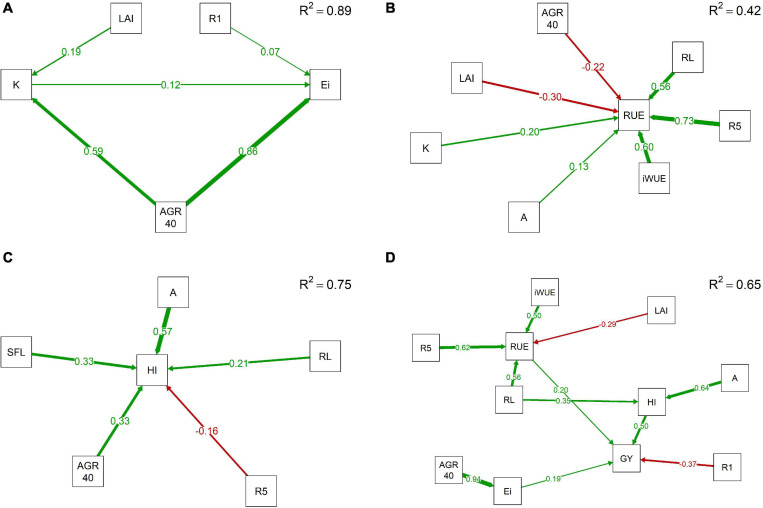
Directed models through path analyses for additive–genetic relationship among physiological and phenological traits with light interception efficiency (*Ei*) **(A)**, radiation use efficiency (*RUE*) **(B)**, harvest index (*HI*) **(C)**, and grain yield (*GY*) **(D)** in a maturity-controlled panel of soybean. Three hundred eighty-three recombinant inbred lines (RILs) evaluated in three environments. *A*, photosynthesis; *AGR40*, average canopy coverage growth rate during the first 40 *DAP*; *K*, light extinction coefficient; *iWUE*, intrinsic water use efficiency; *LAI*, leaf area index; *R1*, days to flowering; *R5*, days to beginning of seed formation; *R8*, days to maturity; *RL*, reproductive period length; *SFL*, seed-filling length.

The efficiency of light interception is mainly determined by the average canopy coverage growth rate during the first 40 days of the growing season with a path coefficient of 0.86 (Figure 1A). Other variables influencing *Ei* include days to *R1* and *K* with path coefficients of 0.07 and 0.12, respectively. *AGR40* along with *LAI* control *K* showing path coefficients of 0.59 and 0.19. *RUE* is positively influenced by days to seed beginning, intrinsic water use efficiency, reproductive length, light extinction coefficient, and photosynthesis (Figure 1B). Path coefficients for these associations varied from 0.73 to 0.13 with high values for *R5*, *iWUE*, and *RL* primarily. An increase of one standard unit of *R5* or *RL* augments 0.73 and 0.56 standard units of *RUE*, respectively. In contrast, *LAI* and *AGR40* negatively influence *RUE* with reduction of −0.30 and −0.22 standard units in *RUE* when one standard unit of *LAI* or *AGR40* is increased, respectively. The average canopy coverage growth rate during the first 40 *DAP* also showed a positive effect in *HI* with a coefficient of 0.33.

Apparent harvest index is highly influenced by photosynthesis, length of seed-filling period, average canopy coverage growth rate during the first 40 days, reproductive length, and days to *R5* (Figure 1C). All these variables are positively related to *HI* except by *R5* with a negative path coefficient of 0.16. Photosynthesis presented the highest path coefficient for *HI* with 0.57; thus, an increase in one standard unit of *A* would produce a positive change in 0.57 standard units of *HI*. *SFL* and *AGR40* also positively contribute to *HI*, where a change of one standard unit of either *SFL* or *AGR40* produces an augment of 0.33 standard units on *HI*. The lowest path coefficient was observed for *RL* with 0.21. Grain yield was positively associated with harvest index, radiation use efficiency, and light interception efficiency with path coefficients of 0.50, 0.20, and 0.19, respectively. Thus, a change in one standard unit of *HI* promotes an increase in 0.50 standard units in *GY*. Contrarily, days to flowering negatively influenced the grain yield in soybean, showing a path coefficient of 0.37 (Figure 1D). Trends in the general model were kept, with *A* and *RL* influencing *HI* and *AGR40* explaining changes in *Ei*; while *RL*, *R5*, *iWUE*, and *LAI* were the main variables affecting *RUE*.

**FIGURE 2:**
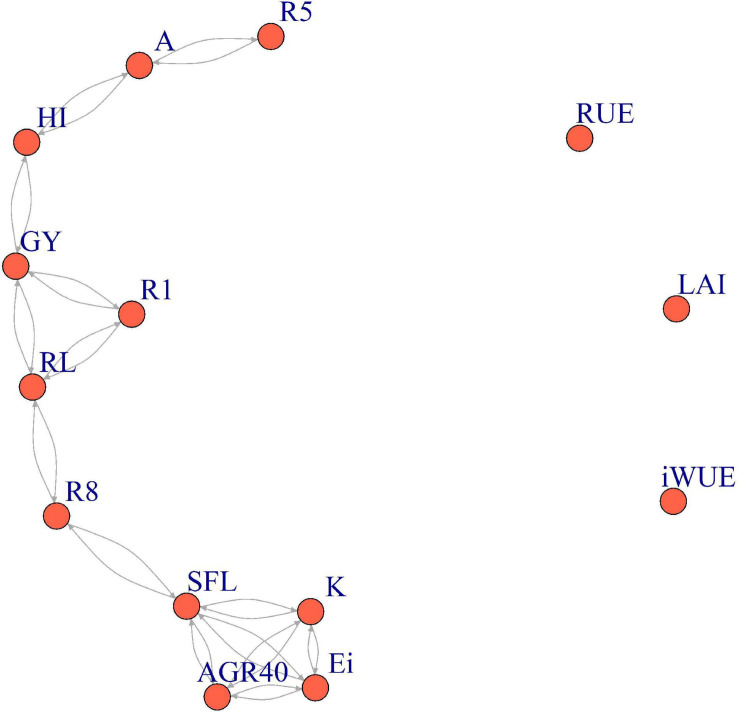
Undirected model through the *LASSO* algorithm for additive–genetic relationship among physiological and phenological traits with light interception efficiency (*Ei*), radiation use efficiency (*RUE*), harvest index (*HI*), and grain yield (*GY*) in a maturity-controlled panel of soybean. Three hundred eighty-three recombinant inbred lines (RILs) evaluated in three environments. *A*, photosynthesis; *AGR40*, average canopy coverage growth rate during the first 40 *DAP*; *K*, light extinction coefficient; *iWUE*, intrinsic water use efficiency; *LAI*, leaf area index; *R1*, days to flowering; *R5*, days to beginning of seed formation; *R8*, days to maturity; *RL*, reproductive period length; *SFL*, seed-filling length; LASSO, least absolute shrinkage and selection operator.

The undirected model (Figure 2) showed a straight influence of *RL*, *R1*, and *HI* in final *GY*, while *HI* is directly associated with photosynthesis. This diagram also depicts the relationship between *RL*, *R1*, and *R8*, with the last phenological stage connected to a node mainly associated with light interception through the variables *SFL*, *Ei*, *AGR40*, and *K*. *RUE*, *LAI*, and *iWUE* were not clustered with other traits through this undirected methodology.

Finally, environmental stability was high for harvest index with Kendall ranking correlation ranging from 0.54 to 0.77 (Table 4). Light interception efficiency showed a high correlation for the locations evaluated during 2018 with a value of 0.82 but a limited correlation when we compared 2017 and 2018 environments. Radiation use efficiency, in turn, presented a moderate correlation when compared with environments from the same location through the years but poor stability between different locations in different years.

**TABLE 4:** Trait stability assessed through the Kendall additive–genetic correlation between the same traits evaluated in different environments for light interception efficiency (*Ei*), radiation use efficiency (*RUE*), and harvest index (*HI*) in a maturity-controlled panel of soybean.

Trait	Environment	ACRE_2017	ACRE_2018	RMN_2018
*Ei*	ACRE_2017	1.00		
	ACRE_2018	–0.45	1.00	
	RMN_2018	–0.49	0.82	1.00
*RUE*	ACRE_2017	1.00		
	ACRE_2018	0.36	1.00	
	RMN_2018	–0.41	–0.07	1.00
*HI*	ACRE_2017	1.00		
	ACRE_2018	0.54	1.00	
	RMN_2018	0.68	0.77	1.00

*Three hundred eighty-three recombinant inbred lines (RILs) evaluated in three environments.*

**FIGURE 3:**
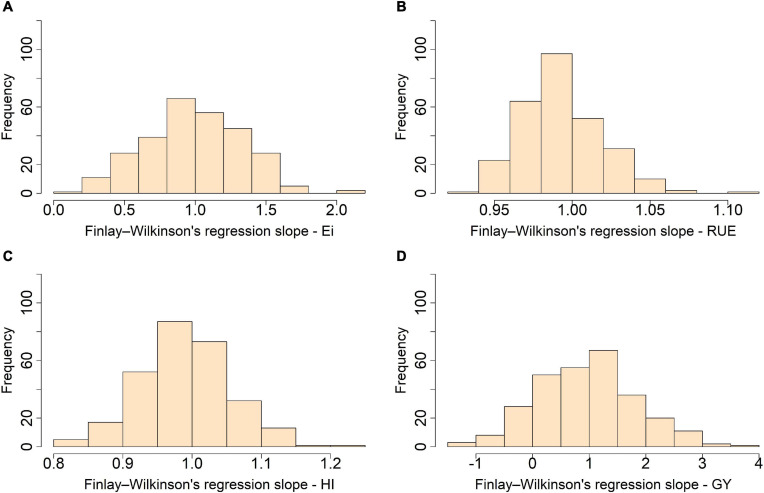
Trait stability evaluated through the distribution for the slope from Finlay and Wilkinson joint regression for light interception efficiency (Ei) **(A)**, radiation use efficiency (RUE) **(B)**, harvest index (HI) **(C)**, and grain yield (GY) **(D)** in a maturity-controlled panel of soybean. Two hundred eighty-one recombinant inbred lines (RILs) evaluated in three environments.

When the stability was assessed through the slopes from the joint regression including not only an additive–genetic effect but also epistasis and the reduced dominance remaining, we found a moderate-to-high stability for *Ei*, *RUE*, and *HI* with distributions centered at 1.0 and narrow interquartile range (IQR) of 0.48, 0.02, and 0.09, respectively (Figure 3). Grain yield, in turn, showed medium-to-low stability with minimum and maximum values of −1.3 and 3.6 and IQR of 1.1.

## Discussion

Path analysis is a multivariate methodology closely related to multivariate regression where the path coefficients correspond to standardized regression coefficients for the linear model suggested by the path diagram (Walsh and Lynch, 1998). The efficiency of light interception is directly affected by canopy architecture and function (Bai et al., 2016; Chavarria et al., 2017). Changes in one standard unit of *AGR40* are associated with changes in 0.86 standard units of *Ei*. Our results indicate that the efficiency of light interception is mainly a function of how fast the canopy develops during the first stages rather than the maximum *LAI* achieved. This results also points out the importance of agronomic decisions affecting early canopy development such as distance between plants, distance between rows, and planting date (Shibles and Weber, 1966; Westgate et al., 1997; Andrade et al., 2002; Edwards et al., 2005) as viable strategies to maximize light interception. Additionally, because of the indirect relationship of *AGR40* and *GY* in the integrate path diagram, it is suggested that capitalizing in early light captured not only increased *Ei* but also might improve grain yield (Figure 1D). The positive effect of canopy coverage rate on grain yield is in accordance with previous reports in soybean and corn (Luque et al., 2006; Xavier et al., 2017a). Light extinction coefficient also influenced *Ei*, since according to equation (2), *K* directly participates in the determination of the amount of solar light remaining after passing through layers of *LAI* (de Wit, 1965; Impens and Lemeur, 1969; Wang et al., 2007; Zhang et al., 2014). Therefore, greater *K* averages suggest planophile canopies with higher light attenuation, while solar radiation passes through the leaves. However, high *K* may also imply less light interception in the lower third of the canopy and probably less canopy photosynthesis as demonstrated by Chen et al. (1994), who showed that upright leaves produced up to 25% higher canopy photosynthesis compared with planophile canopies. Our results are also coherent with the previous finding of Duursma et al. (2012) who described light interception through a simple model involving crown density and leaf dispersion, two variables analogous to canopy coverage and light extinction coefficient. *LAI* plays an indirect role in *Ei* through its influence in *K* that is explained by the multiplicative effect of *LAI* and *K* in equation (2). Thus, greater *LAI* augments the number of layers that light must pass through, increasing the likelihood of solar radiation trapped by the leaves.

Radiation use efficiency is considered the physiological trait that will be the focus for new increases in grain yield to bridge the gap between current and potential values (Melis, 2009; Payne et al., 2012; Reynolds et al., 2012). *RUE* indicates the capability to transform solar radiation, a free resource, into biomass through the plant metabolism. Our results indicate that this efficiency is mainly associated with phenological traits. Longer reproductive length has been previously associated with higher grain yield in soybean (Xavier et al., 2017a), which, along with extended *R5*, would allow to create stronger sources with extra photosynthates to later being translocated to pods and grains (Board and Harville, 1993; Board and Tan, 1995; Board and Kahlon, 2011). Intrinsic water use efficiency, even more than photosynthesis (∼4-fold), was also positively associated with *RUE*, indicating that high photosynthetic rates alone are not enough to produce high biomass per unit of light intercepted. High *iWUE* can reduce the loss of carbon fixation under short water deprivation events (Blankenagel et al., 2018), limiting the *RUE* decrease. In soybean, *iWUE* demonstrated independent variation for both photosynthesis and stomatal conductance with variation mainly attributed to changes in stomatal conductance rather than photosynthesis (Gilbert et al., 2011). Reduction in the seasonal *RUE* and *GY* in soybean is reported as a consequence of water stress during the pod initiation and seed filling (De Costa and Shanmugathasan, 2002; Adeboye et al., 2016). When water deprivation occurs, crop growth rate and dry matter production are reduced as a consequence of a net assimilation decrease mediated by the lack of CO_2_ coming into the leaf (Board and Kahlon, 2011). Likewise, increased daily saturation vapor pressure deficit, a key variable controlling transpiration, is reported as a factor for reducing *RUE* in sorghum and maize even under well-watered conditions (Stockle and Kiniry, 1990). The importance of considering water dynamic in conjunction with carbon metabolism is also pointed out by Wu et al. (2019), who conclude that the impact of enhancing photosynthesis on yield is strongly dependent on the degree of water limitation. These authors suggest modeling the photosynthesis–stomatal conductance relationship as a key factor to better quantify theoretical impacts of improving photosynthesis. The influence of *AGR40* and *LAI* is explained by their direct and indirect contribution to the cumulative light intercepted (Figure 1A), which corresponds to the denominator of *RUE*. These negative associations are also a consequence of the nonlinear relationship between light intercepted and biomass produced when larger amounts of light are intercepted (Edwards et al., 2005). Thus, under greater *LAI* that is likely promoted by high *AGR40*, the soybean cannot maintain a constant rate of biomass production per new amount of light intercepted, diminishing the overall *RUE*. The asymptotic effect of 90% of total biomass in soybean was reported by Edwards et al. (2005), suggesting that any extra light intercepted above 911 MJ m^–2^ would produce a marginal augment of up to 10% in total biomass, with even increases of just 5% when *PAR* intercepted changed from 911 to 1142 MJ m^–2^. Reduction in *LAI* might contribute to enhance *RUE* in soybean, and its feasibility is not completely discarded since it has been demonstrated that 1/3 defoliation does not affect yield and quality as long as *LAI* is above 3.0 (Board and Harville, 1993; Liu et al., 2008).

Harvest index is an indication of reproductive effort with a large contribution on grain yield achievements in cereals during the last decades, especially after the “green revolution” (Donald and Hamblin, 1976; Hay, 1995; Evans et al., 1999; Sadras and Lawson, 2011). Our results show a strong relationship between *HI* and *A*, *SFL*, and *RL*, with *A* being the most remarkable, with increases in one standard unit of *A* promoting changes in 0.57 standard units in *HI*. Augmented *HI* in soybean seems to be a priority to improve grain yield. Achieving this challenge can be made using different approaches, including semi-determinate cultivars through introgression of genes *DT1* and *DT2*, which are presented as promising high-yielding materials with less aboveground vegetative biomass (Kato et al., 2019). Increased seed weight through a higher photosynthetic rate, an extended seed-filling period, and augmented seed size is also proposed as a viable strategy. Photosynthesis is the main process accounting for carbon fixation and, along with respiration, controls the carbohydrates available for grain filling (Board and Kahlon, 2011; Taiz et al., 2014). Extended filling period along with high CO_2_ fixation rates are suggested as synergic events boosting the grain yield formation in soybean. Increased partitioning of carbohydrates is associated with better seed set in soybean (Board and Kahlon, 2011; Rotundo et al., 2012), as the availability of photosynthates during the filling period determines if the seed growing is sink or source limited, with sink limitation occurring when photosynthesis increases and source limitation when photosynthesis is reduced (Egli and Bruening, 2001). The strong contribution to final *GY* from *HI* aligns with reports in wheat, where a significant positive correlation between photosynthesis traits, *HI*, and *GY*, is documented (Foulkes et al., 2011; Xiao et al., 2012; Carmo-Silva et al., 2017). In soybean, in turn, a recent study showed a high genetic correlation between *A* and *GY* (Lopez et al., 2019). The importance of the combination of photosynthesis and duration of the reproductive stage was also demonstrated by Boerma and Ashley (1988), who reported a high correlation of 0.78 between *GY* and the product canopy apparent photosynthesis by seed filling period. Augmented light interception during early stages in soybean increases both number of nodes and number of pods, with the positive effect not only in *HI* but also in *GY* (Board et al., 1992; Board and Tan, 1995). The number of pods per reproductive node was reported as the main yield component in soybean when a path analysis was carried out back in 1999 (Board et al., 1999), whereas a high genetic correlation between early canopy development and *GY* is reported in soybean (Xavier et al., 2017a, b). Days to *R5* showed a negative moderate effect in *HI*, which is explained by the direct effect of extended *R5* in the seed-feeling period considering that the panel we evaluated is maturity-controlled. Progress to increase reproductive length should focus on reducing the time required to flowering since increasing time to maturity involves the logistic problem associated with changes in the maturity group. Phenotypic variation for days to *R1* exists since the data set we collected showed a range of variation for *R1* from 13 to 20 days being as early as 34 days after planting (Table 2). Although flowering in soybean is under the control of photoperiod, temperature, irradiance, and eight “E” genes (Hadley et al., 1984; Cober et al., 2014), insensitive genotypes “day neutral” have been identified (Criswell and Hume, 1972; Polson, 1972; Nissly et al., 1981; Shamugasundaram, 1981; Islam et al., 2019), suggesting that cultivars with less sensitivity to photoperiod might be produced with a theoretical positive effect on *GY*.

Despite that the unsupervised method cannot establish a direction and contribution value for each interaction, the graphical model based on the *LASSO* algorithm revealed most of the relationship we found through the path analysis. The *LASSO* method not only minimizes the residual sum of squares but also constrains some coefficients to exactly zero, performing a parallel variable selection (Tibshirani, 1996). Thus, the absence of connection between *RUE*, *iWUE*, and *LAI* with the full graphical model might be a consequence of overall weak correlations for each of these three traits, with most of the other variables (Table 2) making the algorithm to minimize their contribution to the whole model. In the case of *RUE*, the lack of clustering can be associated also with the moderate inconsistency on the ranking of RILs among the environments mainly promoted by changes in location (ACRE vs. RMN). These changes in ranking, GxE, found for *RUE* contrast with the low sensitivity to variations in location and year showed by *HI* and, along with the low dispersion of the FWR slope, suggest high stability and fewer requirements of multi-environment trials during the *HI* determination. Light interception coefficients, in turn, are strongly influenced by changes in the canopy among years but highly correlated among locations. Differences among years in this study may be explained by particular responses of lines to planting dates since ACRE_2017 was planted late (May 31) compared with ACRE_2018 (May 22) and RMN_2018 (May 17). A negative effect of late planting in *LAI* is reported for soybean (Parvez et al., 1989; Tagliapietra et al., 2018) with a detrimental effect in grain yield also (Egli and Bruening, 1992, 2000; Boote et al., 1998; Egli and Cornelius, 2009).

Genotype × environment (GxE) stability is a desirable performance when new cultivars are released (Bondari, 2003). In soybean, Xavier et al. (2018) recently reported seven genomic regions located on chromosomes 4, 6, 9, 13, 15, and 18 contributing to the GxE response. Likewise, another single region linked to yield stability on chromosome 18 was also documented. In this study, two high-yielding environments (ACRE_2018 and RMN_2018) characterized by extended light harvesting period through higher number of degree days to harvest, well distributed rainfall, and higher mean temperature during the growing season were presented (Table 1). In contrast, a single low-yielding environment (ACRE_2017) associated with reduced growing season for late planting and lower temperature was classified. Our results present stability evaluated through the slope of the FWR (Finlay and Wilkinson, 1963) revealing better stability for *Ei*, *RUE*, and *HI* than *GY* per se. High stability for harvest index in determinate and indeterminate soybean evaluated in the south (Gainesville, FL) and north of the United States (Ithaca, NY) is already reported, aligning with our findings (Spaeth et al., 1984). According to the stability classification, the three efficiencies we assessed showed stability type II, meaning the response to the environment is the same as the mean response with the regression’s slope equal to 1 (Bernardo, 2002). In this case in particular, type II stability suggests that high adaptation to the environment evaluated aligns with the original goal of the SoyNAM population: “improve the yield potential of soybean varieties” with main focus on the maturity group (MG) III^1^. We observed less stability for *GY* denoted through the wide distribution of the slopes around the center with 25% (70) of the RILs showing slopes >1.5, suggesting stability type III, better performance than the average in favorable environments but less than average in unfavorable environments (Bernardo, 2002). On the contrary, 32% (89) showed a probable stability type IV for *GY* with slope < 0.5, implying better than the average response in unfavorable environments but less than the average performance in favorable environments (Bernardo, 2002). Fifty percent (22) of the lines with suggested type III stability come from families classified as high yielding under drought conditions, whereas 26% (28) and 15% (20) have diverse ancestry and high-yielding genetic background, respectively. From the lines with proposed stability type IV, 46% (60) derive from the high-yielding background, 25% (27) come from families with diverse ancestry, and less than 1% (2) come from high yielding under drought. Our results suggest that the material originally bred for environments with water limitations also performs well in favorable environments as observed by Ceccarelli (2015) in barley. In this case, the genetic background for tolerance to water deficit did not impose a penalty to compete in such considerable good environments. Recombinants with high-yielding genetic background respond better to environments considered “unfavorable,” indicating that high-yielding genetic background confers advantages in a wide range of environments.

## Conclusion

Directed and undirected methodologies are able to capture the main relationships underlying light interception efficiency, radiation use efficiency, harvest index, and grain yield, bringing new insights to strategically approach the breeding of complex traits. Advances in soybean productivity must encompass optimization in phenological and physiological processes where improvement on harvest index appears as a suitable strategy to achieve fast and significant advances in final grain yield. Breeding strategies to increase photosynthesis and water use efficiency are a priority because of their positive impact not only in harvest index but also in radiation use efficiency. Although extending the reproductive period length without affecting the total length cycle would require reducing the photoperiod sensitivity and probably increasing the tolerance to cold temperature during the early stages, this phenological improvement has a potential return in the overall soybean perform involving grain yield, harvest index, and radiation use efficiency. Trait stability for individual efficiencies accounting for grain yield, evaluated through the joint regression’s slope, is higher than the stability for grain yield itself, which represent an advantage if selecting for *Ei*, *RUE*, or *HI* was implemented.

## Data Availability Statement

The datasets presented in this study can be found in online repositories. The names of the repository/repositories and accession number(s) can be found at https://www.soybase.org/SoyNAM/index.php.

## Author Contributions

ML and KR conceived and designed the experiments. ML and FF collected the field data. ML conducted the data analysis and interpretation, wrote and edited the manuscript. KR coordinated–supervised the research and ensured the funding. All authors contributed to the article and approved the submitted version.

## Conflict of Interest

FF was employed by company Bayer, Inc. The remaining authors declare that the research was conducted in the absence of any commercial or financial relationships that could be construed as a potential conflict of interest.

## Footnotes

1 https://www.soybase.org/SoyNAM/

## Supplementary Material

Supplementary Figure 1 Comparative for yield and maturity between the phenology-controlled panel and the full Soy-NAM panel.

Supplementary Table 1 Name, class, and program of origin for the 32 families evaluated.

Supplementary Table 2 List of recombinant inbred lines evaluated including family and class.

Supplementary Table 3 Soil fertility information based on soil analyses reports.

Supplementary Table 4 Water balance for the location ACRE_2017.

Supplementary Table 5 Water balance for the location ACRE_2018.

Supplementary Table 6 Water balance for the location RMN_2018.

## References

[B1] Adeboye O. B. Schultz B. Adekalu K. O. Prasad K. (2016). Impact of water stress on radiation interception and radiation use efficiency of Soybeans (*Glycine max* L. Merr.) in Nigeria. *Braz. J. Sci. Technol.* 3:15. 10.1186/s40552-016-0028-1

[B2] Ainsworth E. A. Yendrek C. R. Skoneczka J. A. Long S. P. (2012). Accelerating yield potential in soybean: potential targets for biotechnological improvement. *Plant. Cell Environ.* 35 38–52. 10.1111/j.1365-3040.2011.02378.x 21689112

[B3] Andrade F. H. Calviño P. Cirilo A. Barbieri P. (2002). Yield responses to narrow rows depend on increased radiation interception. *Agron. J.* 94 975–980. 10.2134/AGRONJ2002.9750

[B4] Bai Z. Mao S. Han Y. Feng L. Wang G. Yang B. (2016). Study on light interception and biomass production of different cotton cultivars. *PLoS One* 11:e0156335. 10.1371/journal.pone.0156335 27227675 PMC4882027

[B5] Balboa G. R. Sadras V. O. Ciampitti I. A. (2018). Shifts in soybean yield, nutrient uptake, and nutrient stoichiometry: a historical synthesis-analysis. *Crop Sci.* 58:43. 10.2135/cropsci2017.06.0349

[B6] Bastidas A. M. Setiyono T. D. Dobermann A. Cassman K. G. Elmore R. W. Graef G. L. (2008). Soybean sowing date: the vegetative, reproductive, and agronomic impacts. *Agron. Hortic.* 48 727–740. 10.2135/cropsci2006.05.0292

[B7] Bates D. Vazquez A. I. Ana M. Vazquez I. (2015). *Package “pedigreemm” – Pedigree-Based Mixed-Effects Models.* Available online at: http://pedigreemm.r-forge.r-project.org/ (accessed July 30, 2019).

[B8] Bernardo R. (2002). *Breeding for Quantitative Traits in Plants*, 2 Edn, ed. Bernardo R. (Woodbury, MN: Stemmapress).

[B9] Blankenagel S. Yang Z. Avramova V. Schön C.-C. Grill E. (2018). Generating plants with improved water use efficiency. *Agronomy* 8:194. 10.3390/agronomy8090194

[B10] Board J. E. Harville B. G. (1993). Soybean yield component responses to a light interception gradient during the reproductive period. *Crop Sci.* 33:772. 10.2135/cropsci1993.0011183X003300040028x

[B11] Board J. E. Kahlon C. S. (2011). *Soybean Physiol. Biochem*, ed. El-Shemy H. (Rijeka: InTech Open). 10.5772/1006

[B12] Board J. E. Kamal M. Harville B. G. (1992). Temporal importance of greater light interception to increased yield in narrow-row soybean. *Agron. J.* 84:575. 10.2134/agronj1992.00021962008400040006x

[B13] Board J. E. Kang M. S. Harville B. G. (1999). Path analyses of the yield formation process for late-planted soybean. *Agron. J.* 91:128. 10.2134/agronj1999.00021962009100010020x

[B14] Board J. E. Tan Q. (1995). Assimilatory capacity effects on soybean yield components and pod number. *Crop Sci.* 35:846. 10.2135/cropsci1995.0011183X003500030035x

[B15] Boerma H. R. Ashley D. A. (1988). Canopy photosynthesis and seed-fill duration in recently developed soybean cultivars and selected plant introductions. *Crop Sci.* 28:137. 10.2135/cropsci1988.0011183X002800010029x

[B16] Bogue A. G. (1983). Changes in mechanical and plant technology: the corn belt, 1910-1940. *J. Econ. Hist.* 43 1–25. 10.1017/S0022050700028953

[B17] Bondari K. (1990). “Path analysis in agricultural research,” in *Conference on Applied Statistics in Agriculture*, Vol. 14, (Manhattan). 10.4148/2475-7772.1439

[B18] Bondari K. (2003). “Statistical analysis of genotype X environment interaction in agricultural research,” in *SESUG: The Proceedings of the SouthEast SAS Users Group, Paper SD15* (St. Pete Beach), 7.

[B19] Boote K. J. Jones J. W. Hoogenboom G. Pickering N. B. (1998). “The CROPGRO model for grain legumes,” in *Understanding Options for Agricultural Production. Systems Approaches for Sustainable Agricultural Development*, eds Suji G. Y. Hoogenboom G. Thornton P. K. (Dordrecht: Springer), 99–128. 10.1007/978-94-017-3624-4_6

[B20] Carmo-Silva E. Andralojc P. J. Scales J. C. Driever S. M. Mead A. Lawson T. (2017). Phenotyping of field-grown wheat in the UK highlights contribution of light response of photosynthesis and flag leaf longevity to grain yield. *J. Exp. Bot.* 68 3473–3486. 10.1093/jxb/erx169 28859373 PMC5853948

[B21] Carter T. E. Nelson R. L. Sneller C. H. Cui Z. (2004). “Genetic diversity in soybean,” in *Soybeans: Improvement, Production, and Uses*, eds Boerma H. R. Specht J. E. (Madison, WI: American Society of Agronomy), 303–416.

[B22] Ceccarelli S. (2015). Efficiency of plant breeding. *Crop. Sci.* 55:87. 10.2135/cropsci2014.02.0158

[B23] Chavarria G. Caverzan A. Müller M. Rakocevic M. (2017). *Soybean Architecture Plants: From Solar Radiation Interception to Crop Protection Soybean – The Basis of Yield, Biomass and Productivity.* London: Intech Open, 15–33. 10.5772/67150

[B24] Chen S. G. Shao B. Y. Impens I. Ceulemans R. (1994). Effects of plant canopy structure on light interception and photosynthesis. *J. Quant. Spectrosc. Radiat. Transf.* 52 115–123. 10.1016/0022-4073(94)90144-9

[B25] Cober E. Curtis D. Stewart D. Morrison M. (2014). Quantifying the effects of photoperiod, temperature and daily irradiance on flowering time of soybean isolines. *Plants* 3 476–497. 10.3390/plants3040476 27135515 PMC4844283

[B26] Cregan P. B. Jarvik T. Bush A. L. Shoemaker R. C. Lark K. G. Kahler A. L. (1999). An integrated genetic linkage map of the soybean genome. *Crop. Sci.* 39 1464–1490.

[B27] Criswell J. G. Hume D. J. (1972). Variation in sensitivity to photoperiod among early maturing soybean strains. *Crop. Sci.* 12:657. 10.2135/cropsci1972.0011183X001200050031x

[B28] De Bruin J. L. Pedersen P. (2009). Growth, yield, and yield component changes among old and new soybean cultivars. *Agron. J.* 101:187. 10.2134/agronj2008.0187

[B29] De Costa W. A. J. M. J. M. Shanmugathasan K. N. (2002). Physiology of yield determination of soybean (*Glycine max* (L.) Merr.) under different irrigation regimes in the sub-humid zone of Sri Lanka. *F. Crop. Res.* 75 23–35. 10.1016/S0378-4290(02)00003-5

[B30] de Wit C. T. (1965). Photosynthesis of leaf canopies. *Agric. Res. Rep.* 5 1–54. 10.2172/4289474 4289474

[B31] Donald C. M. Hamblin J. (1976). The biological yield and harvest index of cereals as agronomic and plant breeding criteria. *Adv. Agron.* 59 361–405. 10.1016/S0065-2113(08)60559-3

[B32] Duursma R. A. Falster D. S. Valladares F. Sterck F. J. Pearcy R. W. Lusk C. H. (2012). Light interception efficiency explained by two simple variables: a test using a diversity of small- to medium-sized woody plants. *N. Phytol.* 193 397–408. 10.1111/j.1469-8137.2011.03943.x 22066945

[B33] Edwards J. T. Purcell L. C. Karcher D. E. (2005). Soybean yield and biomass responses to increasing plant population among diverse maturity groups: II. Light interception and utilization. *Crop. Sci.* 45 1778–1785. 10.2135/cropsci2004.0570

[B34] Egli D. B. (2008). Comparison of corn and soybean yields in the United States: historical trends and future prospects. *Agron. J.* 100 S–79. 10.2134/agronj2006.0286c

[B35] Egli D. B. Bruening W. (1992). Planting date and soybean yield: evaluation of environmental effects with a crop simulation model: SOYGRO. *Agric. For. Meteorol.* 62 19–29. 10.1016/0168-1923(92)90003-M

[B36] Egli D. B. Bruening W. P. (2000). Potential of early-maturing soybean cultivars in late plantings. *Agron. J.* 92 532–537. 10.2134/agronj2000.923532x

[B37] Egli D. B. Bruening W. P. (2001). Source-sink relationships, seed sucrose levels and seed growth rates in soybean. *Ann. Bot.* 88 235–242. 10.1006/anbo.2001.1449

[B38] Egli D. B. Cornelius P. L. (2009). A regional analysis of the response of soybean yield to planting date. *Agron. J.* 101 330–335. 10.2134/agronj2008.0148

[B39] Epskamp S. Stuber S. Nak J. Veenman M. (2019). *Path Diagrams and Visual Analysis of Various SEM Packages’ Output*, Vol. 34. Available online at: https://github.com/SachaEpskamp/semPlot (accessed July 3, 2019).

[B40] Evans L. T. Fisher R. A. Fischer R. A. (1999). Yield potential: its definition, measurement, and significance. *Crop Sci. Soc. Am.* 39 1544–1551. 10.2135/cropsci1999.3961544x

[B41] Evans R. G. Sadler E. J. (2008). Methods and technologies to improve efficiency of water use. *Water Resour. Res* 44:W00E04. 10.1029/2007WR006200

[B42] Fehr W. R. Caviness C. E. (1977). *Stages of Soybean Development.* Available online at: http://lib.dr.iastate.edu/specialreports/87 (accessed September 12, 2018).

[B43] Fehr W. R. Caviness C. E. Burmood D. T. Pennington J. S. (1971). Stage of development descriptions for soybeans, *Glycine max* (L.) Merrill. *Crop. Sci.* 11:929. 10.2135/cropsci1971.0011183x001100060051x

[B44] Finlay K. W. Wilkinson G. N. (1963). The analysis of adaptation in a plant-breeding programme. *Aust. J. Agric. Res.* 14 742–754. 10.1071/AR9630742

[B45] Foulkes M. J. Slafer G. A. Davies W. J. Berry P. M. Sylvester-Bradley R. Martre P. (2011). Raising yield potential of wheat. III. Optimizing partitioning to grain while maintaining lodging resistance. *J. Exp. Bot.* 62 469–486. 10.1093/jxb/erq300 20952627

[B46] Fox C. M. Cary T. R. Colgrove A. L. Nafziger E. D. Haudenshield J. S. Hartman G. L. (2013). Estimating soybean genetic gain for yield in the northern united states—influence of cropping history. *Crop Sci.* 53:2473. 10.2135/cropsci2012.12.0687

[B47] Gardner B. L. (2002). *American Agriculture in the Twentieth Century: How it Flourished and What it Cost*, 1 Edn. Cambridge, MA: Harvard University Press.

[B48] Giannakas K. Yiannaka A. (2004). The market potential of a new high-oleic soybean: an ex ante analysis. *AgBioForum* 7 101–112.

[B49] Gilbert M. E. Zwieniecki M. A. Holbrook N. M. (2011). Independent variation in photosynthetic capacity and stomatal conductance leads to differences in intrinsic water use efficiency in 11 soybean genotypes before and during mild drought. *J. Exp. Bot.* 62 2875–2887. 10.1093/jxb/erq461 21339387

[B50] Grassini P. Specht J. E. Tollenaar M. Ciampitti I. Cassman K. G. (2015). High-yield maize-soybean cropping systems in the US Corn Belt. *Crop Physiol.* 15 17–41. 10.1016/B978-0-12-417104-6.00002-9

[B51] Hadley P. Roberts E. H. Summerfield R. J. Minchin F. R. (1984). Effects of temperature and photoperiod on flowering in soya bean [*Glycine max* (L.) Merrill]: a quantitative model. *Ann. Bot.* 53 669–681. 10.1093/oxfordjournals.aob.a086732

[B52] Hartwig E. E. (1973). “Varietal development,” in *Soybeans?: Improvement, Production and Uses*, ed. Caldwell B. E. (Madison, WI: American Society of Agronomy), 187–207.

[B53] Hastie T. Tibshirani R. Friedman J. (2009). *The Elements of Statistical Learning.* Berlin: Springer. 10.1007/b94608

[B54] Hay R. K. M. (1995). Harvest index: a review of its use in plant breeding and crop physiology. *Ann. Appl. Biol.* 126 197–216. 10.1111/j.1744-7348.1995.tb05015.x

[B55] Hearst A. (2019). *Remote Sensing of Soybean Canopy Cover, Color, and Visible Indicators of Moisture Stress Using Imagery from Unmanned Aircraft Systems.* Available online at: https://hammer.figshare.com/articles/Remote_Sensing_of_Soybean_Canopy_Cover_Color_and_Visible_Indicators_of_Moisture_Stress_Using_Imagery_from_Unmanned_Aircraft_Systems/8023478 (accessed June 22, 2019).

[B56] Heatherly L. G. Elmore R. W. (2004). “Managing inputs for peak production,” in *Soybeans: Improvement, Production, and Uses*, eds Boerma H. R. Specht J. E. (Madison, WI: ASA), 451–536. 10.2134/agronmonogr16.3ed.c10

[B57] Impens I. Lemeur R. (1969). Extinction of net radiation in different crop canopies. *Arch. Meteorol. Geophys. Bioklimatologie Ser. B* 17 403–412. 10.1007/BF02243377

[B58] Islam M. R. Fujita D. Watanabe S. Zheng S.-H. (2019). Variation in photosensitivity of flowering in the world soybean mini-core collections (GmWMC). *Plant Prod. Sci.* 22 220–226. 10.1080/1343943X.2018.1561197

[B59] Johnson R. R. (1987). “Management,” in *Soybeans: Improvement, Production, and Uses*, ed. Wilcox J. R. (Madison, WI: ASA), 355–390.

[B60] Kato S. Sayama T. Taguchi-Shiobara F. Kikuchi A. Ishimoto M. Cober E. (2019). Effect of change from a determinate to a semi-determinate growth habit on the yield and lodging resistance of soybeans in the northeast region of Japan. *Breed. Sci.* 69 151–159. 10.1270/jsbbs.18112 31086493 PMC6507727

[B61] Kendall M. G. (1938). A new measure of rank correlation. *Biometrika* 30:81. 10.2307/2332226

[B62] Koester R. P. Skoneczka J. A. Cary T. R. Diers B. W. Ainsworth E. A. (2014). Historical gains in soybean (*Glycine max* Merr.) seed yield are driven by linear increases in light interception, energy conversion, and partitioning efficiencies. *J. Exp. Bot.* 65 3311–3321. 10.1093/jxb/eru187 24790116 PMC4071847

[B63] Kumudini S. Hume D. J. Chu G. (2001). Genetic improvement in short season soybeans: I. Dry matter accumulation, partitioning, and leaf area duration. *Crop Sci.* 41 391–398. 10.2135/cropsci2002.1410 11756264

[B64] Kusmec A. Srinivasan S. Nettleton D. Schnable P. S. (2017). Distinct genetic architectures for phenotype means and plasticities in Zea mays. *Nat. Plants* 3 715–723. 10.1038/s41477-017-0007-7 29150689 PMC6209453

[B65] Lado B. Matus I. Rodríguez A. Inostroza L. Poland J. Belzile F. (2013). Increased genomic prediction accuracy in wheat breeding through spatial adjustment of field trial data. *G3 Genes Genom. Genet.* 3 2105–2114. 10.1534/g3.113.007807 24082033 PMC3852373

[B66] Lian L. de los Campos G. (2016). FW: an R package for Finlay–Wilkinson regression that incorporates genomic/pedigree information and covariance structures between environments. *G3 Genes Genom. Genet.* 6 589–597. 10.1534/g3.115.026328 26715095 PMC4777122

[B67] LI-COR Inc (2012). *LAI-2200 Plant Canopy Analyzer*, 1 Edn. Lincoln, NE: LI-COR Inc. 10.1016/B978-1-4832-1312-5.50007-9

[B68] Liu X. Jin J. Wang G. Herbert S. J. (2008). Soybean yield physiology and development of high-yielding practices in Northeast China. *F. Crop. Res.* 105 157–171. 10.1016/j.fcr.2007.09.003

[B69] Lopez M. A. Xavier A. Rainey K. M. (2019). Phenotypic variation and genetic architecture for photosynthesis and water use efficiency in soybean (*Glycine max* L. Merr). *Front. Plant Sci.* 10:680. 10.3389/fpls.2019.00680 31178887 PMC6543851

[B70] Luedders V. D. (1977). Genetic improvement in yield of soybeans. *Crop Sci.* 17:971. 10.2135/cropsci1977.0011183X001700060040x

[B71] Luque S. F. Cirilo A. G. Otegui M. E. (2006). Genetic gains in grain yield and related physiological attributes in Argentine maize hybrids. *F. Crop. Res.* 95 383–397. 10.1016/j.fcr.2005.04.007

[B72] Meinshausen N. Bühlmann P. (2006). High-dimensional graphs and variable selection with the Lasso. *Ann. Stat.* 34 1436–1462. 10.1214/009053606000000281

[B73] Melis A. (2009). Solar energy conversion efficiencies in photosynthesis: Minimizing the chlorophyll antennae to maximize efficiency. *Plant Sci.* 177 272–280. 10.1016/j.plantsci.2009.06.005

[B74] Monsi M. Saeki T. (1953). Uber den Lichtfaktor in den Pflanzengesellschaf- u ur die Stoffproduktion. *Japanese J. Bot.* 14 22–52.

[B75] Monteith J. L. (1972). Solar radiation and productivity in tropical ecosystems. *J. Appl. Ecol.* 9 747–766. 10.2307/2401901

[B76] Monteith J. L. (1977). Climate and the efficiency of crop production in Britain. *Philos. Trans. R. Soc. Lond.* 281 277–294.

[B77] Morrison M. J. Voldeng H. D. Cober E. R. (2000). Agronomic changes from 58 years of genetic improvement of short-season soybean cultivars in Canada. *Agron. J.* 92:780. 10.2134/agronj2000.924780x

[B78] Murphy K. (2014). “Machine learning: a probabilistic perspective,” in *Machine Learning*, (Cambridge, MA: MIT Press), 661–705. Available online at: https://www.cs.ubc.ca/~murphyk/MLbook/pml-print3-ch19.pdf (accessed July 20, 2019).

[B79] Nissly C. R. Bernard R. L. Hittle C. N. (1981). Variation in photoperiod sensitivity for time of flowering and maturity among soybean strains of maturity group III. *Crop Sci.* 21:833. 10.2135/cropsci1981.0011183X002100060009x

[B80] NRCS (2018). *Web Soil Survey.* Washington, DC: Natural Resources Conservation Service. 10.3389/fimmu.2013.00258

[B81] Parvez A. Q. Gardner F. P. Boote K. J. (1989). Determinate- and indeterminate-type soybean cultivar responses to pattern, density, and planting date. *Crop Sci.* 29 150–157. 10.2135/cropsci1989.0011183X002900010034x

[B82] Payne T. Reynolds M. Skovmand B. (2012). “Searching genetic resoures for useful variation in physiological traits,” in *Physiological Breeding I: Interdisciplinary Approaches to Improve Crop Adaptation*, eds. Reynolds M. P. Pask A. Mullan D. (Mexico: CIMMYT), 51–59.

[B83] Petzoldt T. (2018). *Package Version 0.8.2 ‘growthrates’: Estimate Growth Rates from Experimental Data*, Vol. 39. 10.1093/molbev/mst197 24132122 PMC3840312

[B84] Polson D. E. (1972). Day-neutrality in soybeans. *Crop Sci.* 12:773. 10.2135/cropsci1972.0011183X001200060017x

[B85] Probst A. H. Judd R. W. (1973). “Origin, U.S. history and development and world distribution,” in *Soybeans: Improvement, Production and Uses*, Vol. 16 ed. Caldwell B. E. (Madison, WI: American Society of Agronomy), 1–15.

[B86] Purcell L. C. (2000). Soybean canopy coverage and light interception measurements using digital imagery. *Crop Sci.* 40 834–837. 10.2135/cropsci2000.403834x

[B87] R Core team (2019). *R Core Team.* Vienna: R Foundation for Statistical Computing.

[B88] Reddy K. N. (2001). Glyphosate-resistant soybean as a weed management tool: opportunities and challenges. *Weed Biol. Manag*. 1, 193–202. 10.1046/j.1445-6664.2001.00032.x

[B89] Reynolds M. P. Hellin J. Govaerts B. Kosina P. Sonder K. Hobbs P. (2012). Global crop improvement networks to bridge technology gaps. *J. Exp. Bot.* 63 1–12. 10.1093/jxb/err241 21926090

[B90] Rincker K. Nelson R. Specht J. Sleper D. Cary T. Cianzio S. R. (2014). Genetic improvement of U.S. soybean in maturity groups II, III, and IV. *Crop Sci.* 54 1419–1432. 10.2135/cropsci2013.10.0665

[B91] Rosseel Y. (2012). lavaan: an R package for structural equation mdeling. *J. Stat. Softw.* 48 1–36. 10.18637/jss.v048.i02

[B92] Rotundo J. L. Borrás L. De Bruin J. Pedersen P. (2012). Physiological strategies for seed number determination in soybean: biomass accumulation, partitioning and seed set efficiency. *F. Crop. Res.* 135 58–66. 10.1016/j.fcr.2012.06.012

[B93] Rowntree S. C. Suhre J. J. Weidenbenner N. H. Wilson E. W. Davis V. M. Naeve S. L. (2013). Genetic gain x management interactions in soybean: I. Planting date. *Crop Sci.* 53 1128–1138. 10.2135/cropsci2012.03.0157

[B94] Rowntree S. C. Suhre J. J. Weidenbenner N. H. Wilson E. W. Davis V. M. Naeve S. L. (2014). Physiological and phenological responses of historical soybean cultivar releases to earlier planting. *Crop Sci.* 54 804–816. 10.2135/cropsci2013.06.0428

[B95] Sadras V. O. Lawson C. (2011). Genetic gain in yield and associated changes in phenotype, trait plasticity and competitive ability of South Australian wheat varieties released between 1958 and 2007. *Crop Pasture Sci.* 62 533–549. 10.1071/CP11060

[B96] Sakurai G. Iizumi T. Nishimori M. Yokozawa M. (2014). How much has the increase in atmospheric CO2 directly affected past soybean production? *Sci. Rep.* 4 4978–4982. 10.1038/srep04978 24827887 PMC4021318

[B97] Shamugasundaram S. (1981). Varietal differences and genetic behaviour for the photoperiodic responses in soybeans. *Bull. Inst. Trop. Agric. Kyushu Univ.* 4 1–6.

[B98] Shibles R. M. Weber C. R. (1966). Interception of solar radiation and dry matter production by various soybean planting patterns. *Crop Sci.* 6:55. 10.2135/cropsci1966.0011183X000600010017x

[B99] Spaeth S. C. Randall H. C. Sinclair T. R. Vendeland J. S. (1984). Stability of soybean harvest index. *Agron. J.* 76:482. 10.2134/agronj1984.00021962007600030028x

[B100] Specht J. E. Diers B. W. Nelson R. L. Francisco J. de Toledo F. Torrion J. A. (2014). “Soybean,” in *Yield Gains in Major U.S. Field Crops*, eds S. Smith, B. W. Diers, J. E. Specht, and B. Carver (Madison, WI: American Society of Agronomy, Inc., Crop Science Society of America, Inc., and Soil Science Society of America, Inc.), 311–356. 10.2135/cssaspecpub33.c12

[B101] Specht J. E. Hume D. J. Kumudini S. V. (1999). Soybean yield potential – A genetic and physiological perspective. *Crop Sci.* 39:1560. 10.2135/cropsci1999.3961560x

[B102] Specht J. E. Williams J. H. (1984). “Contribution of genetic technology to soybean productivity — retrospect and prospect,” in *Genetic Contributions to Yield Gains of Five Major Crop Plants*, (Madison, WI: Crop Science Society of America), 49–74. 10.2135/cssaspecpub7.c3

[B103] Steinsland I. Jensen H. (2010). Utilizing gaussian Markov random field properties of Bayesian animal models. *Biometrics* 66 763–771. 10.1111/j.1541-0420.2009.01336.x 19817739

[B104] Stockle C. O. Kiniry J. R. (1990). Variability in crop radiation-use efficiency associated with vapor-pressure deficit. *F. Crop. Res.* 25 171–181. 10.1016/0378-4290(90)90001-R

[B105] Suhre J. J. Weidenbenner Nicholas H. Rowntree S. C. Wilson E. W. Naeve S. L. Conley S. P. (2014). Soybean yield partitioning changes revealed by genetic gain and seeding rate interactions. *Agron. J.* 106 1631–1642. 10.2134/agronj14.0003

[B106] Tagliapietra E. L. Streck N. A. Da Rocha T. S. M. Richter G. L. Da Silva M. R. Cera J. C. (2018). Optimum leaf area index to reach soybean yield potential in subtropical environment. *Agron. J.* 110 932–938. 10.2134/agronj2017.09.0523

[B107] Taiz L. Zeiger E. Moller I. M. Murphy A. (2014). *Plant Physiology and Development*, 6 Edn. Oxford: Sinauer. 10.3119/0035-4902-117.971.397

[B108] Tibshirani R. (1996). Regression shrinkage and selection via the Lasso. *J. R. Stat. Soc. Ser. B* 58 267–288. 10.1111/j.2517-6161.1996.tb02080.x

[B109] USDA–NASS (2020). *National Statistics for Soybeans.* United States Department. Available online at: https://www.nass.usda.gov/Statistics_by_Subject/result.php?B69543EC-6199-3B8E-B0BA-85AD269C8504&sector=CROPS&group=FIELD CROPS&comm=SOYBEANS (accessed June 24, 2019).

[B110] Ustun A. Allen F. L. English B. C. (2001). Genetic progress in soybean of the U.S. *Midsouth. Crop Sci.* 41 993–998. 10.2135/cropsci2001.414993x

[B111] Vanous A. Gardner C. Blanco M. Martin-Schwarze A. Wang J. Li X. (2019). Stability analysis of kernel quality traits in exotic-derived doubled haploid maize lines. *Plant Genome* 12:114. 10.3835/plantgenome2017.12.0114 30951103 PMC12962363

[B112] Voldeng H. D. Cober E. R. Hume D. J. Gillard C. Morrison M. J. (1997). Fifty-eight years of genetic improvement of short-season soybean cultivars in Canada. *Crop Sci.* 37:428. 10.2135/cropsci1997.0011183X003700020020x

[B113] Walsh J. B. Lynch M. (1998). “Measuring multivariate selection,” in *Genetics and Analysis of Quantitative Traits*, Sinauer, 370–391. Available online at: http://nitro.biosci.arizona.edu/courses/EEB600A/download/Chapter_20.pdf (accessed July 5, 2019).

[B114] Wang W. M. Li Z. L. Su H. B. (2007). Comparison of leaf angle distribution functions: effects on extinction coefficient and fraction of sunlit foliage. *Agric. For. Meteorol.* 143 106–122. 10.1016/j.agrformet.2006.12.003

[B115] Westgate M. E. Forcella F. Reicosky D. C. Somsen J. (1997). Rapid canopy closure for maize production in the northern US corn belt: radiation-use efficiency and grain yield. *F. Crop. Res.* 49 249–258. 10.1016/S0378-4290(96)01055-6

[B116] Wilcox J. R. Schapaugh W. T. Bernard R. L. Cooper R. L. Fehr W. R. Niehaus M. H. (1979). Genetic improvement of soybeans in the midwest. *Crop Sci.* 19:803. 10.2135/cropsci1979.0011183X001900060014x

[B117] Wilson E. W. Rowntree S. C. Suhre J. J. Weidenbenner N. H. Conley S. P. Davis V. M. (2014). Genetic gain × management interactions in soybean: II. Nitrogen utilization. *Crop Sci.* 54:340. 10.2135/cropsci2013.05.0339

[B118] Wright S. (1960). Path coefficients and path regressions alternative complemetary concepts? *Biometrics* Available online at: https://about.jstor.org/terms (accessed July 20, 2019).

[B119] Wu A. Hammer G. L. Doherty A. von Caemmerer S. Farquhar G. D. (2019). Quantifying impacts of enhancing photosynthesis on crop yield. *Nat. Plants* 5 380–388. 10.1038/s41477-019-0398-8 30962528

[B120] Xavier A. Hall B. Casteel S. Muir W. Rainey K. M. (2017a). Using unsupervised learning techniques to assess interactions among complex traits in soybeans. *Euphytica* 213:200. 10.1007/s10681-017-1975-4

[B121] Xavier A. Hall B. Hearst A. A. Cherkauer K. A. Rainey K. M. (2017b). Genetic architecture of phenomic-enabled canopy coverage in Glycine max. *Genetics* 206 1–15. 10.1534/genetics.116.198713 28363978 PMC5499164

[B122] Xavier A. Jarquin D. Howard R. Ramasubramanian V. Specht J. E. Graef G. L. (2018). Genome-wide analysis of grain yield stability and environmental interactions in a multiparental soybean population. *G3 Genes Genom. Genet.* 8:g3.300300.2017. 10.1534/g3.117.300300 29217731 PMC5919731

[B123] Xavier A. Xu S. Muir W. M. Rainey K. M. (2015). NAM: association studies in multiple populations. *Bioinformatics* 31 3–4. 10.1093/bioinformatics/btv448 26243017

[B124] Xiao Y. G. Qian Z. G. Wu K. Liu J. J. Xia X. C. Ji W. Q. (2012). Genetic gains in grain yield and physiological traits of winter wheat in Shandong province, China, from 1969 to 2006. *Crop Sci.* 52 44–56. 10.2135/cropsci2011.05.0246

[B125] Zhang L. Hu Z. Fan J. Zhou D. Tang F. (2014). A meta-analysis of the canopy light extinction coefficient in terrestrial ecosystems. *Front. Earth Sci.* 8:599–609. 10.1007/s11707-014-0446-7

[B126] Zhao T. Liu H. Roeder K. Lafferty J. Wasserman L. (2012). The huge package for high-dimensional undirected graph estimation in R. *J. Mach. Learn. Res.* 13 1059–1062. 10.1002/aur.1474.Replication 26834510 PMC4729207

