# Supplementary figures and images for: Genetic Relationships Among Physiological Processes, Phenology, and Grain Yield Offer an Insight Into the Development of New Cultivars in Soybean (Glycine max L. Merr)
Source: Front Plant Sci. 2021 Apr 9;12:651241. doi: 10.3389/fpls.2021.651241 (PMC8064921; doi:10.3389/fpls.2021.651241)

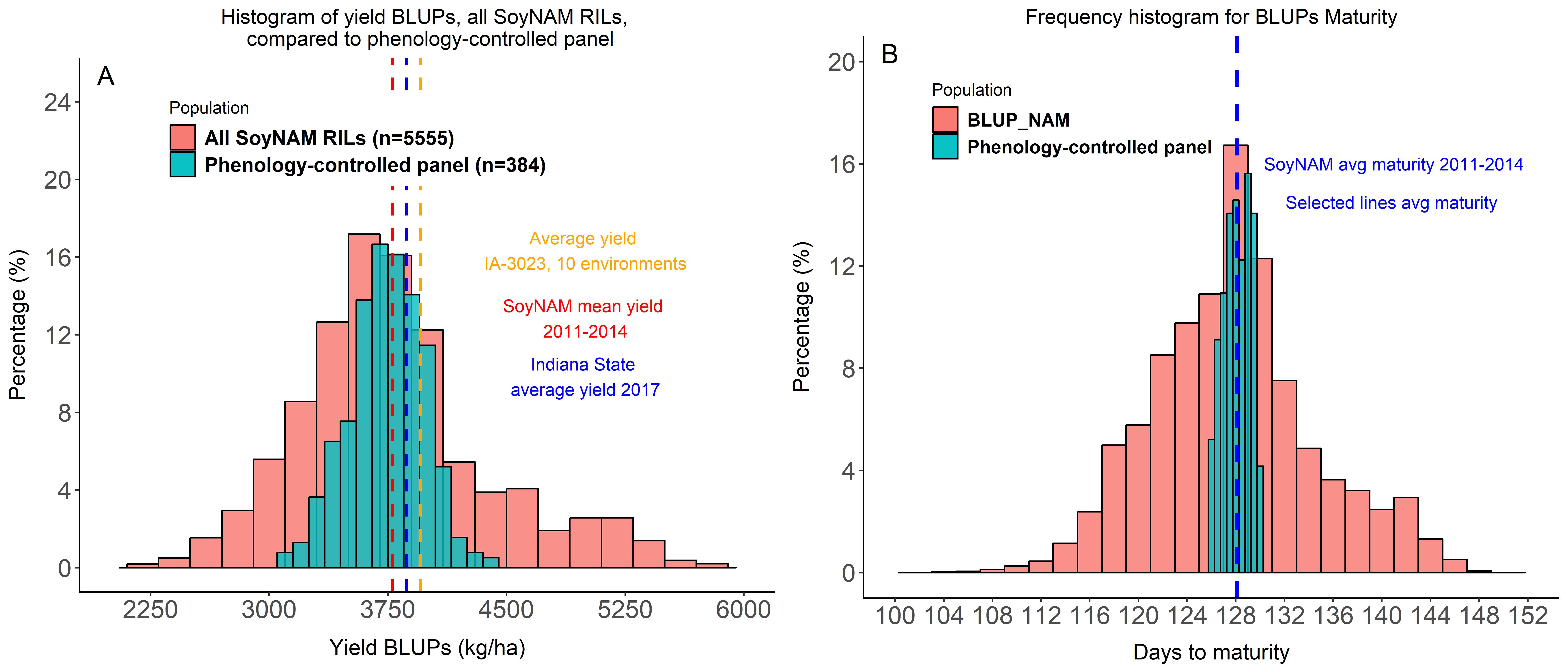

Supplement: Supplementary Figure 1 — Comparative for yield and maturity between the phenology-controlled panel and the full Soy-NAM panel. [file Image_1.JPEG]
